# Detection of ALDH3B2 in Human Placenta

**DOI:** 10.3390/ijms20246292

**Published:** 2019-12-13

**Authors:** Sylwia Michorowska, Joanna Giebułtowicz, Renata Wolinowska, Anna Konopka, Anna Wilkaniec, Paweł Krajewski, Ewa Bulska, Piotr Wroczyński

**Affiliations:** 1 Department of Bioanalysis and Drug Analysis, Faculty of Pharmacy, Medical University of Warsaw, 02-097 Warsaw, Poland; joanna.giebultowicz@wum.edu.pl (J.G.); piotr.wroczynski@wum.edu.pl (P.W.); 2 Department of Pharmaceutical Microbiology, Centre for Preclinical Research and Technology (CePT), Faculty of Pharmacy, Medical University of Warsaw, 02-097 Warsaw, Poland; renata.wolinowska@wum.edu.pl; 3 Biological and Chemical Research Centre, Faculty of Chemistry, University of Warsaw, 02-097 Warsaw, Poland; a.konopka@cnbc.uw.edu.pl (A.K.); ebulska@chem.uw.edu.pl (E.B.); 4 Department of Cellular Signaling, Mossakowski Research Centre, Polish Academy of Sciences, Pawińskiego 5, 02-106 Warsaw, Poland; awilkaniec@imdik.pan.pl; 5 Forensic Medicine Department, First Faculty of Medicine, Medical University of Warsaw, 02-097 Warsaw, Poland; pawel.krajewski@wum.edu.pl

**Keywords:** *ALDH3B2*, premature stop codon, placenta, readthrough

## Abstract

Aldehyde dehydrogenase 3B2 (*ALDH3B2*) gene contains a premature termination codon, which can be skipped or suppressed resulting in full-length protein expression. Alternatively, the longest putative open reading frame starting with the second in-frame start codon would encode short isoform. No unequivocal evidence of ALDH3B2 expression in healthy human tissues is available. The aim of this study was to confirm its expression in human placenta characterized by the highest *ALDH3B2* mRNA abundance. *ALDH3B2* DNA and mRNA were sequenced. The expression was investigated using western blot. The identity of the protein was confirmed using mass spectrometry (MS). The predicted tertiary and quaternary structures, subcellular localization, and phosphorylation sites were assessed using bioinformatic analyses. All DNA and mRNA isolates contained the premature stop codon. In western blot analyses, bands corresponding to the mass of full-length protein were detected. MS analysis led to the identification of two unique peptides, one of which is encoded by the nucleotide sequence located upstream the second start codon. Bioinformatic analyses suggest cytoplasmic localization and several phosphorylation sites. Despite premature stop codon in DNA and mRNA sequences, full-length ALDH3B2 was found. It can be formed as a result of premature stop codon readthrough, complex phenomenon enabling stop codon circumvention.

## 1. Introduction

*ALDH3B2* gene, also known as *ALDH8*, belongs to the aldehyde dehydrogenase (ALDH) gene superfamily [1]. This superfamily consists of 19 putatively functional genes [2] that encode enzymes catalyzing the NAD(P)+ -dependent irreversible oxidation of aldehydes to the corresponding carboxylic acids. The importance of their physiological, detoxification, and protective functions is evidenced by the fact that mutations and polymorphism of *ALDH* genes, resulting in a decreased or lost enzymatic activity, lead to severe diseases such as Sjögren-Larsson syndrome, cancer, and Alzheimer’s disease to name a few [2,3]. The majority of ALDH proteins have been well characterized by now. So far, there is no information available about function of ALDH3B2 isozyme and data related to its expression in healthy human individuals are ambiguous.

The *ALDH3B2* gene located on human chromosome 11q13.2 consists of at least 10 exons [4]. It exhibits high structural similarity to the *ALDH3B1* gene. The numbers and positions of the introns, as well as the boundaries of the individual exons of the two genes, are precisely conserved [4]. The two genes have relatively high nucleotide positional identities in the corresponding coding regions (86%) [5]. However, at the 17th codon (starting from the first start codon) premature in-frame stop codon was identified in the cDNA sequence of *ALDH3B2*. The possibility of it being the product of a variant *ALDH3B2* gene allele was verified by sequencing genomic DNA fragments containing the variant codon isolated from 10 unrelated people of the Asian population by Hsu and Chang [5]. The analysis revealed that all PCR products contained the termination codon, suggesting that *ALDH3B2* gene could be a non-processed pseudogene [5]. However, it was found that seven CpG dinucleotides in *ALDH3B2* gene-related amplicon had decreased mean methylation levels in sperm DNA obtained from males with reduced fecundity in comparison to control (the proven fertile males) [6]. The observed CpG methylation pattern indicates that *ALDH3B2* gene is active and its expression is controlled by an epigenetic mechanism. Depending on the location of CpG sites their hypermethylation can result in either increased (when located in a gene body) or decreased (when located in promoters or enhancers) gene expression [7].

*ALDH3B2* mRNA was found in human salivary glands by Hsu and Chang [5] and in small intestine, colon, kidney, lung, stomach, testis, thymus, thyroid gland, trachea, pancreas, uterus, prostate, salivary gland, and placenta by Nishimura and Naito [8] by reverse transcriptase PCR (RT-PCR) analysis, which indicates that this gene is transcribed. The premature stop codon found in *ALDH3B2* mRNA can be eliminated by alternative splicing, RNA editing [4], or translational recoding.

**Figure 1:**
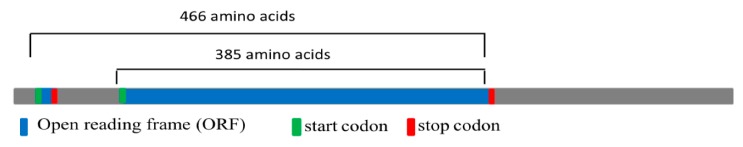
Structure of *ALDH3B2* gene. Blue represents open reading frames (ORF), green represents the start codon, and red represents the stop codon. Black lines show lengths of two ALDH3B2 isoforms: the short one with 385 amino acids and the long one with 466 amino acids.

Regulation of gene expression at the translational level can be achieved via the following three recoding events: stop-codon readthrough, ribosome frameshifting, and translational bypassing. The first phenomenon does not alter the translational reading frame and extends the polypeptide C-terminally [9]. Stop codon can be decoded as a sense codon by either a near-cognate tRNA (tRNA with anticodons that have a single mismatch upon pairing to a stop codon) or the specialized cognate tRNAs (tRNAs with an anticodon that is complementary to the stop codon), such as tRNA^Sec^ [9]. Both of these recoding events would result in the full-length ALDH3B2 expression (466 amino acids (aa), molecular weight around 53 kDa; longer variant shown in Figure 1). Failing to maintain the correct reading frame in the production of protein results in ribosome frameshifting in − or + direction. Spontaneous frameshifting produces non-functional peptides while programmed ribosome frameshifting (which is the frameshifting enhanced by the signals in the mRNA) typically leads to the synthesis of a functional polypeptide from an altered frame. The third mentioned recoding event, translational bypassing, produces a single protein from a discontinuous reading frame. A portion of the mRNA is skipped by the translating ribosome leading to the production of only one polypeptide from a discontinued frame. In case of the *ALDH3B2* gene, the ribosome could translate the first 16 mRNA codons up to the UGA stop codon. Then instead of terminating protein synthesis, the ribosome could slide over a non-coding gap and continue the synthesis after reaching the matching landing codon, which in case of *ALDH3B2* would be first downstream AAG triplet, as the matching take-off and landing codon is the key bypassing signal [9]. Translational bypassing would result in the production of a protein a little bit shorter than the full-length isoform (466 aa), but longer than the short one (385 aa). Alternatively, the translation of *ALDH3B2* transcript containing premature stop codon can be started from the second start codon (that is the first downstream in-frame ATG codon). In case of such a scenario, the protein produced would be 385 aa long (molecular weight around 43 kDa; shorter isoform shown in Figure 1), thus shorter than typical ALDH proteins, but still containing regions important for catalytic functions characteristic of this group of enzymes [5].

Two unique ALDH3B2 peptides were detected in proteomic analysis of forty human lung adenocarcinoma cell lines [10]. These peptides were assigned to short ALDH3B2 isoform, which may indicate that only peptides mapping to protein region encoded by nucleotide sequence located downstream of the second start codon were identified. However, identification of peptides mapping to the sequence of short ALDH3B2 isoform does not provide conclusive evidence of the presence of short ALDH3B2 variant in cell lines subjected to the analysis, as such peptides could originate from either short or long ALDH3B2 isoform. Unfortunately, neither their sequences, nor their mass spectra, are available for evaluation. Moreover, some ALDH isozymes are known to be differently expressed in healthy and cancerous tissues (e.g., ALDH1A3 [11]) and literature data indicate elevated *ALDH3B2* mRNA levels in lung squamous cell carcinoma and lung adenocarcinoma compared to healthy tissues [12]. The only available data about ALDH3B2 protein expression in healthy human tissues can be found in the Human Proteome Map database [13]. Sequences and mass spectra of the following peptides were identified: HLTPVTLELGGK, VTLELGGK, NPCYVDDNCDPQTVANR, FYGDDPQSSPNLGR, YFNAGQTCVAPDYVLCSPEMQER, and LLPALQSTITR [13]. However, careful evaluation revealed that none of them is unique to ALDH3B2. The high sequence homology of ALDH3B1 and ALDH3B2 makes the differentiation between those two isoenzymes extremely challenging.

The *ALDH3B2* DNA sequence analysis results that are available only for Asian population and ambiguous data concerning the detection of ALDH3B2 protein in cell lines and human tissues do not provide conclusive evidence for ALDH3B2 expression in humans. Thus, the aim of this study was to confirm the expression of ALDH3B2 protein in healthy human tissues by western blot and mass spectrometry analyses. Additionally, to shed some light on *ALDH3B2* expression mechanism, sequencing analyses of genomic DNA, and mRNA isolated from Caucasian race individuals were performed. All tests were conducted on placenta samples as this tissue was found to express the highest levels of *ALDH3B2* mRNA as found by Nishimura and Naito [8].

## 2. Results

### 2.1. Recombinant Protein Expression in Escherichia coli and Western Blot Analysis

**Figure 2:**
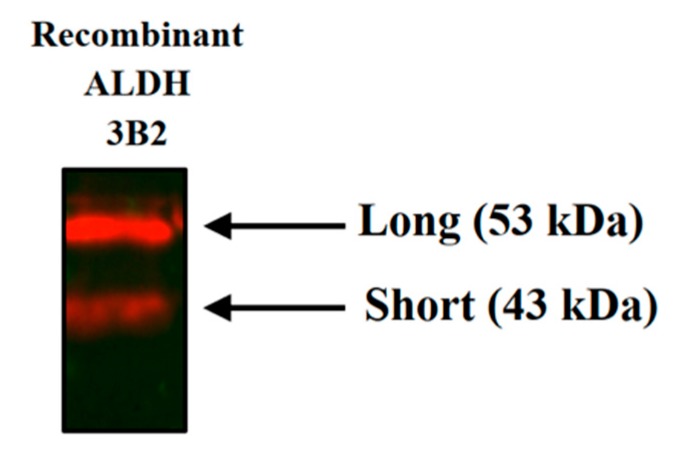
Western blot analysis using anti-ALDH3B2 antibody confirmed the expression of both short and long isoforms of recombinant ALDH3B2 protein in *E. coli*. Purified recombinant proteins were applied onto the polyacrylamide gel. After separation, they were transferred onto the PVDF membrane. Their immunodetection was performed using anti-ALDH3B2 antibody. The image was taken using ChemiDoc XRS+ (Bio-Rad).

Both short and long isoforms of ALDH3B2 protein were obtained using pET28a(+)-*E. coli* expression system (Figure 2). These recombinant proteins were used as positive controls in western blot analyses and enabled the selection of the appropriate gel bands for cutting out in the in-gel digestion protocol used in mass spectrometry analyses.

**Figure 3:**
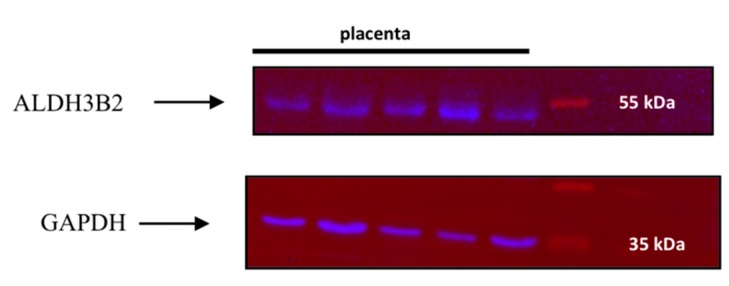
Western blot analysis of placenta homogenates using anti-ALDH3B2 and anti-GAPDH antibodies revealed bands corresponding to the molecular weight of 53 kDa (long isoform of ALDH3B2) and bands corresponding to molecular weight of 37 kDa (GAPDH). Proteins in homogenates were applied onto the polyacrylamide gel. After separation, they were transferred onto the PVDF membrane. Immunodetection of ALDH3B2 protein was performed using anti-ALDH3B2 antibody. After membrane stripping, the immunodetection was repeated with anti-GAPDH antibody. GAPDH detection was used as a loading control, as *GAPDH* gene is constitutively expressed at high levels in many tissues. The image was taken using ChemiDoc XRS+ (Bio-Rad). Additionally, the image of the whole membrane was added into the Supplementary Materials (Supplementary Figure S1).

Western blot analysis of placenta homogenates revealed no bands corresponding to the mass 43 kDa. Surprisingly, bands corresponding to a greater mass, around 53 kDa, were obtained (Figure 3).

### 2.2. DNA and RNA Analyses

**Figure 4:**
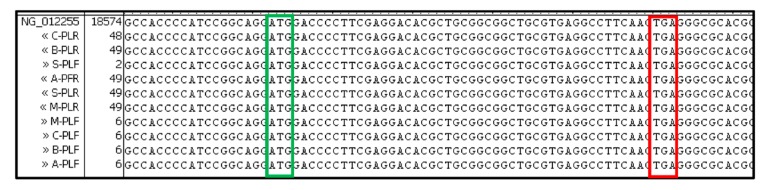
All sequences of *ALDH3B2* DNA isolated from placenta samples contained premature stop codon. Sequence alignment was performed using Vector NTI Advance^TM^ *11.0* software. As a reference *ALDH3B2* sequence from NCBI (NG_012255.1) was used. Green frame shows first start codon and red one premature stop codon.

**Figure 5:**
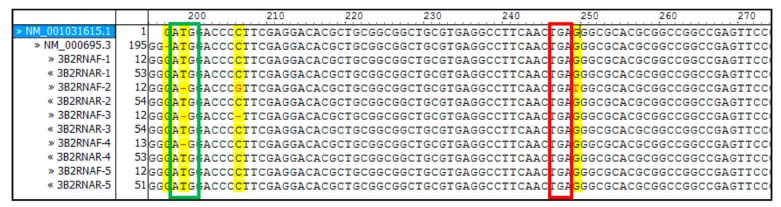
All sequences of *ALDH3B2* cDNA synthesized in RT-PCR reaction using mRNA isolated from placenta samples contained premature stop codon. Sequence alignment was performed using Vector NTI Advance^TM^ *11.0* software. Isolated RNA was treated with DNase to remove contaminating DNA and then subjected to RT-PCR. The obtained cDNA was sequenced. As a reference, *ALDH3B2* cDNA sequences from NCBI (NM_001031615.1 and NM_000695.3) were used. The green frame shows first start codon and red frame shows the premature stop codon. Yellow indicates differences in the aligned sequences.

Genomic DNA isolated from placenta samples as well as *ALDH3B2* mRNA RT-PCR products contained premature stop codon at the 17th codon position downstream the 1st start codon (Figure 4 and Figure 5). No allelic variants were detected.

### 2.3. MS/MS Analysis

Long ALDH3B2 and ALDH3B1 isoforms’ peptides were found among the tryptic fragments of proteins present in placenta homogenates. The identified amino acid sequences were as follows: LLPALQSTITR and FYGDDPQSSPNLGR—peptides common to ALDH3B1 as well as both, short and long ALDH3B2; AAQLQGLGHFLQENK—peptide unique to long ALDH3B2; and LDYIFFTGSPR—a peptide common to long and short ALDH3B2, but not present in the sequence of ALDH3B1.

**Figure 6:**
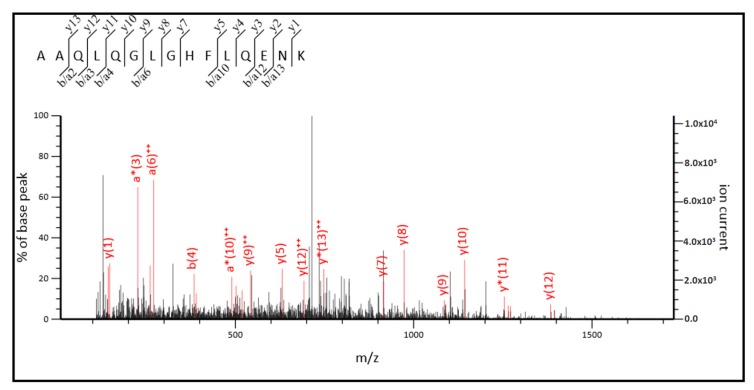
MS/MS spectrum for the tryptic peptide, AAQLQGLGHFLQENK, found in the sequence of long ALDH3B2 isoform. The upper left corner indicates the source of the fragment ions identified as either b, a, or y ions. ^++^ designates doubly charged fragment ions, * designates fragment ions with neutral loss.

**Figure 7:**
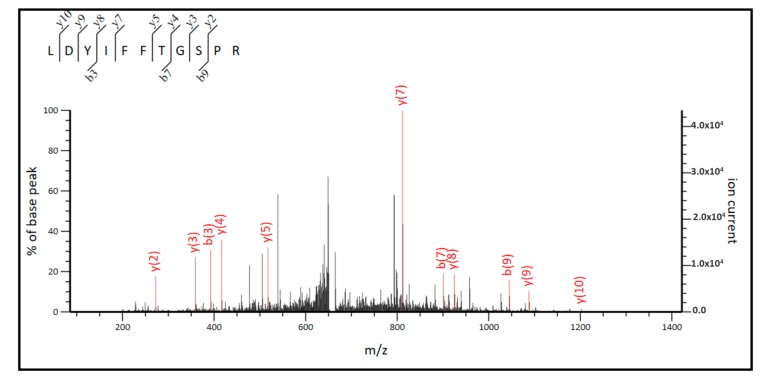
MS/MS spectrum for the tryptic peptide, LDYIFFTGSPR, found in the sequence of long and short ALDH3B2 isoform. The upper left corner indicates the source of the fragment ions identified as either b or y ions.

The MS/MS spectra for the sequencing of two of them, AAQLQGLGHFLQENK and LDYIFFTGSPR, are shown in Figure 6 and Figure 7, respectively.

**Figure 8:**
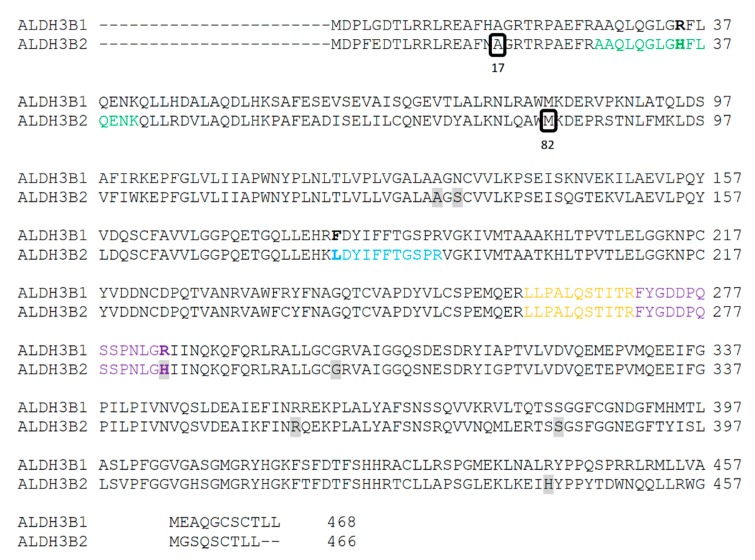
Peptides identified in MS/MS analysis of placenta homogenates. Peptide sequences identified in the MS/MS analysis were mapped to the long ALDH3B2 isoform sequence (translation product of mRNA transcript number U37519.1 from NCBI obtained using ExPASy server [14] with alanine in the position encoded by premature stop codon) and compared with the sequences of the corresponding peptides of ALDH3B1 (NCBI: NP_001154945.1). Colors indicate sequences of identified peptides: yellow and violet—sequences common to ALDH3B1 as well as short and long ALDH3B2; blue—sequence common to both, short and long ALDH3B2 isoforms; and green—sequence unique to long ALDH3B2 isoform. Residues in bold represent amino acids different for ALDH3B1 and ALDH3B2 within sequences of identified peptides. Grey shading indicates positions of different amino acids present in different ALDH3B2 natural variants that can be found in UniProt database [15]: residue 50: A/T, residue 52: S/N, residue 203:H/R, residue 220: G/S, residues 276: R/W, residue 302: S/R, residue 361: H/R. Two black frames in the sequence of ALDH3B2 indicate: the alanine in the position encoded by the premature stop codon (17th aa) and methionine encoded by the first in-frame start codon (82nd aa), respectively.

As it can be observed in Figure 8, three out of four identified peptides correspond to the part of the sequences common to both, short, and long ALDH3B2 isoforms. Among the observed peptides only one is unique to this isoenzyme (**L**DYIFFTGSPR) and differs by only one amino acid when compared to the corresponding sequence of ALDH3B1 (**F**DYIFFTGSPR). The fourth identified peptide (AAQLQGLG**H**FLQENK) is encoded by the nucleotide sequence located upstream the second start codon, indicating the presence of long ALDH3B2 isoform. Its sequence differs by only one amino acid (histidine) from the corresponding sequence of ALDH3B1, AAQLQGLG**R**FLQENK (arginine). Detailed analysis of MS/MS spectrum registered for AAQLQGLG**H**FLQENK peptide (Figure 6) indicates the presence of y7 fragment ion with the amino acid sequence HFLQENK proving the presence of histidine residue in the peptide structure, thus confirming the identification of long ALDH3B2 in the placenta homogenate. The corresponding AAQLQGLG**R**FLQENK sequence characteristic of ALDH3B1 after trypsin digestion would give two short peptides since R is a cleavage site for trypsin. Even if this cleavage site is missed by enzyme, what sometimes happens, m/z registered for this amino acid sequence would be different (the mass difference between H and R residues is 19.0422). In the similar way, peptide sequence **L**DYIFFTGSPR, which is common to the long and short ALDH3B2 differs by one amino acid residue (leucine) from the corresponding ALDH3B1 peptide sequence **F**DYIFFTGSPR (phenylalanine). In MS/MS spectra recorded for **L**DYIFFTGSPR (Figure 7), b3 (LDY), b7 (LDYIFFT), and b9 (LDYIFFTGS) fragment ions were identified, all containing leucine in their structures. This proves the presence of long (and/or short) ALDH3B2 isoform.

As a control experiment, proving the successful use of MS/MS analysis to verify the presence of long ALDH3B2 isoform in placenta homogenate, a recombinant long ALDH3B2 protein was analyzed. Amino acids sequences identified as well as MS/MS spectrum registered for AAQLQGLG**H**FLQENK unique to long ALDH3B2 are presented in Supplementary Materials (Supplementary Figure S3). For the recombinant protein six unique peptides, present in the part of the protein encoded by nucleotides located upstream the second start codon, were identified.

### 2.4. Bioinformatic Analysis

**Figure 9:**
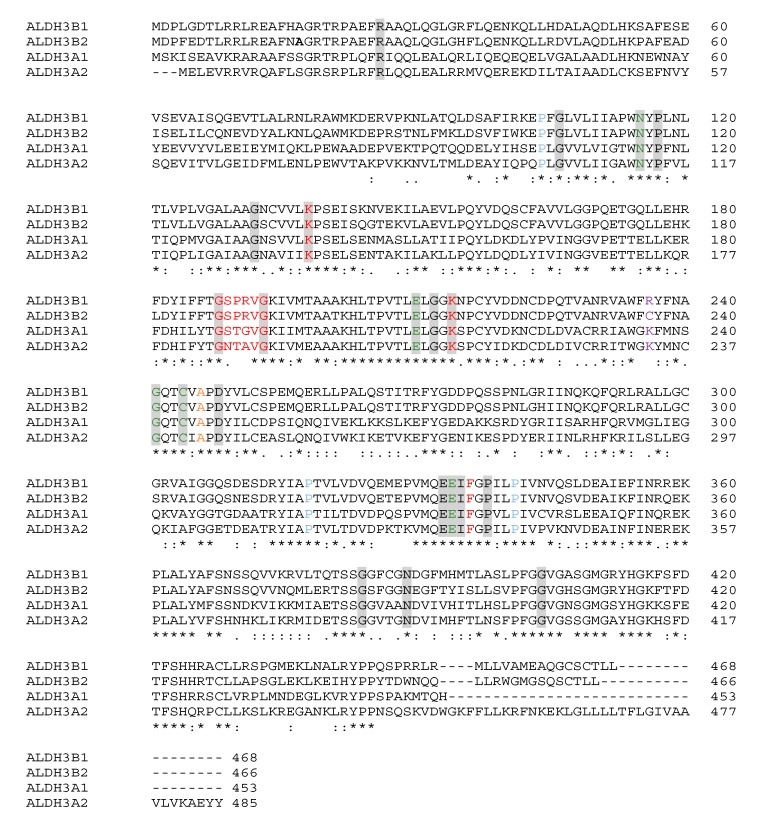
Comparison of ALDH3 isoenzymes’ primary structures. The alignment of ALDH3 sequences was performed using Clustal Omega server [16] and the following sequences: NCBI sequence NP_001128640.1 of ALDH3A1, NCBI sequence NP_000373.1 of ALDH3A2, NCBI sequence NP_001154945.1 of ALDH3B1 and long ALDH3B2 isoform sequence, the product of translation of mRNA transcript number U37519.1 from NCBI obtained using ExPASy server [14], with alanine in the position encoded by premature stop codon. Orange indicates residues involved in substrate binding, red residues involved in cofactor binding, blue three proline residues contributing to the tertiary structure, violet residue 236, gray shading residues highly conserved: Cys-244 acting as a nucleophile; Glu-334 activating thiol group of Cys-244; Gly-241 positioning Cys-244; Glu-210 activating water molecule that hydrolyzes ester group, residues responsible for cofactor binding: Gly-188 and Gly-193 forming Rossmann fold; Lys-138, Glu-334, and Phe-336 responsible for nicotinamide ring positioning and hydrogen bond formation to the NAD(P)^+^ adenine ribose; Ile-335 and Lys-214 ensuring appropriate geometry of cofactor binding domain; Asn-115 crucial for catalytic activity, transiently stabilizing oxygen of carboxyl group of tetrahedral intermediate; Asp-248 conserved in ALDHs belonging to the third subfamily, responsible for appropriate geometry of active site; Arg-26, Gly-106, Pro-117, Gly-132, Pro-338, Gly-384, Asn-389, and Gly-404 [1,17].

The alignment of all ALDH3 isoenzymes’ sequences, including the sequence of long ALDH3B2 isoform with alanine in the position encoded by the premature stop codon, is shown in Figure 9.

Twenty-two residues were found to be highly conserved in ALDH3 subfamily. These include active site residues (Asn-115, Glu-210, Cys-244, Glu-334), 15 glycine residues (e.g., Gly-188 and Gly-212 located within the stem of the active funnel, Gly-188 and Gly-193 forming the Rossmann fold), and three proline residues (Pro-103, Pro-318 and Pro-341 contributing to the tertiary structure) [17]. As it can be observed in Figure 9, all highly conserved residues are present in the sequence of long ALDH3B2 isoform, including Asp-248 being the residue the most indicative of class 3 ALDH. However, attention should be paid to the residue 236, whose function was elucidated in the context of Sjögren-Larsson syndrome. In the case of ALDH3A2, Lys-236 is known to protonate the transient oxyanion formed during the catalysis [1]. Replacing it with cysteine present in this position in ALDH3B2 may affect enzyme activity.

**Figure 10:**
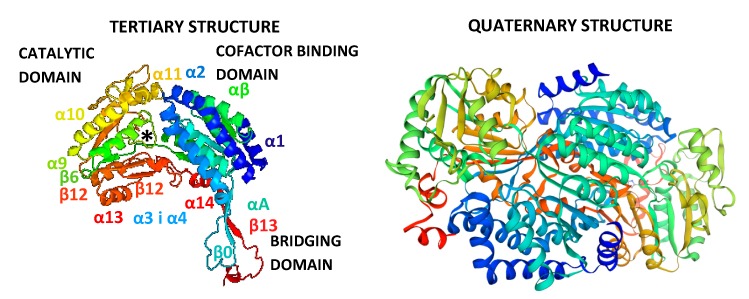
Predicted tertiary and quaternary structure of human long ALDH3B2 isoform, with alanine in the position encoded by the premature stop codon. The tertiary structure model was prepared using Phyre2 server [18] and the quaternary structure model was prepared using Swiss Model server [19]. ALDH3B2 sequence used was the product of translation of mRNA transcript number U37519.1 from NCBI obtained using ExPASy server [14], with alanine in the position encoded by premature stop codon. In both cases, the rat class 3 aldehyde dehydrogenase template at 2.6 Å resolution was used (PDB ID: 1AD3). The confidence was 100%, coverage 95% (444 residues: 4-447) with 50% sequence identity. The rainbow color code describes the predicted 3D structures from N- (blue) to C-termini (red). The designation of the three domains as well as that of alpha helices and beta strands was based on the rat ALDH3A1 structure (PDB ID: 1AD3) [20]. The asterisk indicates the catalytic Cys-244.

The predicted tertiary and quaternary structures of long ALDH3B2 isoform are depicted in Figure 10.

The first tertiary structures of class 2 and 3 ALDH were published in 1996–1997 [20,21] and as of today crystal structures of several human ALDH are described in literature, e.g., ALDH1A1 [22], ALDH1A3 [23], ALDH2 [24], ALDH3A1 [24], 3A2 [25], ALDH4A1 [26]. Among ALDHs both, dimers and tetramers can be found. The predicted tertiary structure for human long ALDH3B2 isoform was compared with rat ALDH3A1. Similar structures were observed including the three major domains: the catalytic domain, the NAD binding domain and an oligomerization domain, involved in the dimer formation (Figure 10). The two first mentioned domains are separated by the active site cleft [1]. The quaternary structure shows the homo-dimer. The Global Model Quality Estimation (GMQE) is 0.77 indicating high reliability. The value of QMEAN Z-score (−0.01) indicates good agreement between the model structure and experimental structures of similar size.

Low probability of transmembrane helices formation by long ALDH3B2 isoform was obtained using TMHMM Server v. 2.0 [27,28] (Supplementary Figure S4). However, another analysis using SOSUI server [29] showed the possibility of two transmembrane helices formation: one between 118 and 140 amino acid residues and second one between 151 and 173 amino acid residues.

The subcellular localization predicted using WoLF PSORT server [30] suggests, that long ALDH3B2 isoform is a cytoplasmic protein, while the sequence analysis performed using NetPhos 3.1 [31] suggested several possible phosphorylation sites.

## 3. Discussion

All genomic DNA samples analyzed in this study confirmed the presence of the premature stop codon in *ALDH3B2* gene in Caucasian population as detected by Hsu and Chang in Asian population [5]. This excludes the possibility of it being the variant gene allele. The presence of the premature stop codon in DNA does not necessarily indicate its presence in mRNA transcripts due to the changes in their sequences introduced by alternative splicing or RNA editing. However, in case of *ALDH3B2* mRNA premature stop codon was identified suggesting at first, that short isoform of protein, if any, is expressed in human tissues. It is interesting though, that in analyzed placenta homogenates band corresponding to the protein with greater mass (around 53 kDa) was detected by western blot analysis.

Taking into account questionable reliability of western blot analysis, the identity of the protein detected was confirmed by MS/MS analysis. Two peptides unique to ALDH3B2 were identified in placenta homogenates, among them one mapping to the region upstream the premature stop codon, indicating the expression of the longer variant. It must be emphasized though, that the sequences of each of the observed peptides have only one amino acid that is different for ALDH3B2 and ALDH3B1. According to the UniProt database [15] ALDH3B1 has no natural variants identified so far, which enables assigning those two peptides, AAQLQGLGHFLQENK and LDYIFFTGSPR, to the long isoform of ALDH3B2 isozyme.

Sequence analysis of long ALDH3B2 isoform confirmed the presence of all highly conserved residues in this family of enzymes. However, the presence of cysteine at 236 position (in place of lysine in case of ALDH3A1 and ALDH3A2, and arginine in case of ALDH3B1) suggests the possibility of altered enzymatic activity, which should be assessed experimentally. Mouse ALDH3B2 located in lipid droplets is characterized by broad substrate specificity including medium- and long-chain aldehydes [32]. However, the primary structure of human and mouse ALDH3B2 are not identical. What is more, the amino acid residue in mouse ALDH3B2 that corresponds to the 236 amino acid position of human ALDH3B2 is arginine. Because of the differences in the primary structure the predictions about substrate specificity using mouse enzyme would be rather far-fetched. Additionally, because of the possibility of phosphorylation of some of ALDH3B2 residues, the activity should be determined experimentally, best using purified human enzyme.

The mass of the detected ALDH3B2 protein corresponds to the mass of the protein encoded by the nucleotide sequence starting with the first ATG codon. Observation of the full-length protein would mean that somehow the premature termination codon identified in mRNA sequence is skipped or suppressed. The process of protein synthesis termination is not 100% efficient [33]. There are several ways in which reading frames can be extended by circumventing stop codons including translational bypassing, in-frame suppression of termination codons and ribosomal frame shifting [34]. The peptides identified by mass spectrometry analyses can exclude the possibility of ribosomal frame shifting to be the only process enabling the premature stop codon elimination in case of *ALDH3B3* mRNA, as this process would produce a protein with different primary structure. During the translational bypassing of *ALDH3B2* mRNA the ribosome would take-off after reaching AAG codon, which precedes the premature UGA codon. The first matching landing codon is located 23 triplets downstream the premature stop codon. The peptide AAQLQGLGHFLQENK identified in MS/MS analysis is encoded by the nucleotide sequence starting with 10th codon downstream the premature stop codon (Supplementary Figure S5), which excludes the possibility of translation bypassing as the mechanism of the recoding event occurring during the expression of *ALDH3B2* gene. It is possible that long ALDH3B2 isoform is produced as a result of spontaneous stop codon readthrough. This phenomenon has been well documented in viruses [35], bacteria, yeast [36], and *Drosophila* [37]. The frequency of the occasional readthrough is low <10^−4^ per stop codon [33,38] but it can be increased up to 0.1–0.3 due to the stimulating effects of sequence and structural elements in the mRNA as well as *trans* factors [39,40]. Moreover, in-frame premature stop codons represent a genotypic subset of mutations that make up ~11% of all known mutations that cause genetic diseases, and millions of patients have diseases attributable to premature termination codons [41].

First example of eukaryotic mammalian readthrough protein was rabbit β-globin, whose mRNA contains premature UGA stop codon [34,42]. Mass spectrometry analysis of isolated rabbit β-globin led to the identification of sequences of seven different proteolytic peptide fragments that were encoded by the same mRNA sequence containing premature stop codon. Four of them were formed as a result of readthrough (with serine, tryptophan, cysteine, and arginine being inserted during the stop codon decoding). The other three lacked an amino acid or amino acids corresponding to the stop codon and/or one or two of the neighboring nucleotides, which indicates that they were produced as a result of translational reading gap [34]. There are two other experimentally confirmed readthrough proteins: longer isoform of myelin protein zero (p0 or MPZ), so called large myelin protein zero (L-MPZ) [43], and vascular endothelial growth factor-A isoform (VEGF-Ax) with unique 22-amino acid C-terminal extension and anti-angiogenic activity expressed in mammalian endothelial cells [40]. In the case of the latter example, the stop codon is decoded as serine [40]. *AGO1* and *MTCH2* genes are authentic readthrough targets identified by bioinformatics analysis and transfection experiments using HEK293 cells [40].

Human selenoproteins are formed by the incorporation of selenocysteine (Sec) dictated by in-frame UGA codon. This complex mechanism requires special *trans*-acting protein factors (Sec insertion sequence binding protein 2 and Sec-specific translation elongation factor) and a *cis*-acting Sec insertion sequence (SECIS) element (a stem-loop structure located immediately downstream of the in-frame UGA codon at which Sec is incorporated) [9,44]. For the decoding of a stop codon by near cognate tRNA, genomic analyses, and profiling studies of human genes have found several potential readthrough candidates [9] some of which were confirmed experimentally. About 4% of malate dehydrogenase is physiologically extended by translational readthrough, with arginine and tryptophan being co-encoded by the UGA stop codon [45]. C-extended isoform of human lactate dehydrogenase is produced as a result of readthrough of UGA-CUA motif [46]. Readthrough of the vitamin D receptor mRNA sequence UGACUAG results in an expression of a protein with 67 amino acid-long C-terminal extension (vitamin D receptor X). This longer proteoform is characterized by a reduced transcriptional response to calcitriol [47]. An interesting example of premature stop codon readthrough is the *LAMA3* gene (R943X/R1159X). In one patient carrying heterozygous nonsense mutation of *LAMA3* gene, it was observed that *LAMA3* mRNA escaped nonsense-mediated decay, which resulted in the expression of full length laminin α3 [48].

The MS/MS data collected in this study do not allow for unequivocal elucidation of the mechanism of premature stop codon elimination in case of *ALDH3B2*. Unfortunately, peptide corresponding to the nucleotide sequence encompassing premature TGA codon was not detected. However, one possibility consistent with the data collected is that ALDH3B2 protein can be produced in the process of the stop codon readthrough resulting from the decoding by near-cognate tRNA.

**Figure 11:**
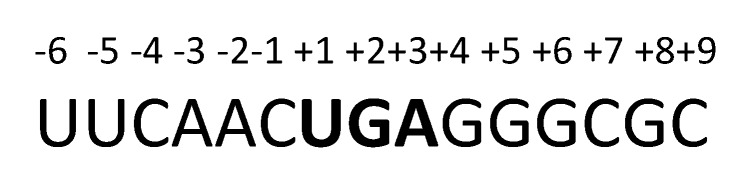
Nucleotide sequence surrounding the premature stop codon in *ALDH3B2* mRNA.

Figure 11 shows the fragment of the *ALDH3B2* mRNA sequence, including premature stop codon.

There are several factors known to affect the efficiency of stop codon readthrough. These include the stop codon itself [49], nucleotide sequence surrounding the stop codon [50], transacting factors [36,51], abundance of near-cognate tRNAs [52], abundance and/or modifications of release factors [53], and the presence of mRNA secondary structures [39,54,55]. Research on mammalian cell lines showed that opal stop codon is characterized by the highest potential of undergoing the process of readthrough [56,57,58,59,60]. Moreover, nucleotides surrounding premature termination codon may interact with nucleotides of the near-cognate acyl-tRNA anticodon loop and consequently stabilize codon–anticodon binding, facilitating the incorporation of an amino acid (Arg, Cys, Trp, Ser, for TGA or Gln for TAG) [34,48,61]. In yeasts, six nucleotides preceding and following the TAG are most influential [62,63]. It was found that readthrough is favored by the presence of two adenines at position -1 and -2 [63]. Other research indicates the substantial role of the nucleotide immediately downstream the stop codon as evidenced by its interaction with the release factors [59], which are involved in the mechanism of translation termination [33]. The nucleotide following the premature termination codon in *ALDH3B2* contains guanine. High readthrough level in experiments using mammalian cell lines was found for cytidine in this position [60]. However, adenine present at -2 position of *ALDH3B2* gene can induce the readthrough by structural modification of mRNA at P-site of ribosome [63]. What is more, in certain cases the deviation from one of the ‘canonical’ readthrough motif may be tolerated [39]. In the case of the *LAMA3* gene mutation R943X, the first and third nucleotides upstream of the premature termination codon or one of the two nucleotides following the premature termination codon had the following consensus sequence (A/T) (A/G) (T/C) TGA CTA. There are also other factors affecting the readthrough. In case of VEGF-Ax, this phenomenon is promoted by the binding of heterogeneous nuclear ribonucleoprotein (hnRNP) A2/B1 (*trans*-factor) to the *cis*-acting element located in the 3′UTR region of the sequence [40]. *Cis*-acting RNA structures can also modulate the readthrough by interfering with release factor recruitment to the ribosome, by modulating ribosome function through the interaction with ribosomal proteins or rRNAs and also by inducing ribosome stalling [9]. In the case of some viruses and *Drosophila* genes, the stimulatory element for efficient readthrough includes a stem-loop structure that spans ~140 nt 3′ of the stop codon. It is possible that this secondary mRNA structure interacts with ribosome directly (pausing it and/or promoting its conformational changes) or provides a physical block that preferentially occludes release factor from the A-site in favor of tRNAs [39]. The complex phenomenon of stop codon decoding by near-cognate tRNA awaits further elucidation.

Sequencing the readthrough protein at the site encoded by the stop codon is crucial to determine whether ALDH3B2 is formed by readthrough or by another mechanism. Helpful would be the overexpression of *ALDH3B2* gene fragment starting with the first start codon and containing the premature stop codon in HEK293 cells, the subsequent purification of the product and its sequence analyses. An alternative way of confirming the readthrough of *ALDH3B2* mRNA could involve the use of aminoglycosides, which are known to induce premature termination codon readthrough in *E. coli,* yeast, and human cultured cells [64].

## 4. Materials and Methods

### 4.1. Preparation of Placenta Homogenates

All procedures were carried out on ice. Dissected and frozen human placenta obtained after Caesarean section of women at the Infant Jesus Teaching Hospital, Warsaw Medical University (*n* = 12) were rinsed with ice cold PBS three times and homogenized manually using 5 mmol dm^−3^ phosphate buffer pH = 7.2 containing 0.25 mol dm^−3^ sucrose and protease inhibitor cocktail (cOmplete^TM^ Mini EDTA-free Protease Inhibitor Cocktail; Roche). To remove chromosomal DNA, cell debris, and fibers, the homogenates were centrifuged at 10,000× *g* for 10 min and the supernatants were collected and stored at −80 °C. The protein concentration was determined using Bradford assay kit (Thermo Scientific^TM^, Waltham, MA, USA) to standardize the amount of protein subjected to gel electrophoresis and subsequent western blot.

### 4.2. Expression of ALDH3B2 in E. coli

In the first step short isoform of recombinant ALDH3B2 (385 aa) was produced using the human *ALDH3B2* gene fragment starting with the second start codon amplified from “TrueClone” cDNA clone in pCMV6-XL5 vector purchased from OriGene. The sequences of the 5′ and 3′ PCR primers were:

5′GGGCTAGCATGAAGGATGAACCACGGTC3′ and

5′GGGAATTCTCACAGGAGGGTGCAGCTC3′ respectively

A *NheI* site was introduced by the PCR primer on the 5′ end, whereas a *EcoRI* site on the 3′ end. The resulting 1.5-kb PCR-amplified fragment was digested with *NheI* and *EcoRI*, gel-purified using the QIAquick Gel Extraction Kit (Qiagen), and ligated with pET-28a(+) vector (Novagene) that has been digested with the same restriction enzymes and gel-purified. The product of the ligation reaction was used to transform *E.coli* BL-21 (DE3) competent cells (Invitrogen). The sequence of the entire insert of the plasmid was verified by sequencing.

The cultures of the overproducing strain were grown at 37 °C in LB broth (35 g/L tryptone, 20 g/L yeast extract, 5 g/L NaCl, Sigma-Aldrich) supplemented with 50 μg/mL kanamycin to an OD_600_ of 0.6. The expression was induced by adding IPTG (isopropyl β-D-1-thiogalactopyranoside, Sigma-Aldrich) to a final concentration of 1 mmol dm^−3^. Recombinant ALDH3B2 was isolated with Ni-NTA Fast Start Kit (Qiagen) and dialyzed overnight to 50 mM pyrophosphate buffer containing 1 mM EDTA and 1 mM DDT (Sigma-Aldrich).

To obtain long ALDH3B2 isoform, the human *ALDH3B2* gene was subjected to mutagenesis to transform the premature stop codon TGA into GCA, codon encoding alanine. The choice of amino acid was dictated by the presence of alanine in the same position in ALDH3B1 protein having the highest sequence homology. The plasmid containing *ALDH3B2* gene fragment starting with the first start codon and containing the premature stop codon was amplified in PCR reaction using two primers phosphorylated at 5′ end with the following sequences:

5′GGCCTTCAAC**G**CAGGGCGCA3′ (bearing point mutation) and

5′TCACGCAGCCGCCGC3′.

There, PCR was performed the following way: 1 cycle of initial denaturation at 98 °C for 30 s, 25 cycles of subsequent denaturation at 98 °C for 10 s, annealing at 72 °C for 15 s, and extension at 72 °C for 30 s, followed by 1 cycle of final extension at 72 °C for 10 min.

The parental methylated and hemimethylated DNA was digested with FastDigest Dpnl (Phusion^TM^Site-Directed Mutagenesis Kit, Thermo Scientific, Waltham, MA, USA) according to the manufacturer’s instructions. The mutated PCR product was circularized by ligation with T4 DNA Ligase (Phusion^TM^Site-Directed Mutagenesis Kit, Thermo Scientific, Waltham, MA, USA). After confirming its sequence, it was used to transform *E. coli.*

### 4.3. Western Blot Analyses

The supernatants obtained after placenta homogenates centrifugation and recombinant proteins samples were denatured and fractionated by sodium dodecyl sulfate-polyacrylamide gel electrophoresis (SDS-PAGE). Separated proteins were transferred onto polyvinylidene difluoride (PVDF) membrane using a transfer apparatus (mini-PROTEAN Tetra Cell, Bio-Rad) according to the manufacturer’s protocol. After incubation with 5% non-fat milk in TBST (10 mmol dm^−3^ Tris, pH 8.0, 150 mmol dm^−3^ NaCl, 0.5% Tween 20) for 60 min, membranes were washed three times with TBST. Human ALDH3B2 protein was detected using goat anti-human ALDH3B2 antibody (1:200, *v/v*) (Santa Cruz Biotechnology, Inc., Dallas, TX, USA) by incubation at 4 °C for 12 h. After being washed three times, membranes were incubated with horseradish peroxidase-conjugated (HRP) anti-goat antibody (1:40,000) (Sigma-Aldrich) for 1 h. Finally, proteins bound with antibody were visualized using chemiluminescence (Pierce ECL Western Blotting Substrate, Thermo Scientific, Waltham, MA, USA) and ChemiDoc XRS+ imaging system (Bio-Rad). Loading of protein samples was confirmed by reprobing membranes with rabbit anti-human glyceraldehyde 3-phosphate dehydrogenase (GAPDH) antibody (1:40,000 (*v/v*)) followed by peroxidase-conjugated anti-rabbit IgG (1:8000, Sigma-Aldrich). The detection of GAPDH also enabled the assessment of the quality of the experiment, e.g., proper transfer of the proteins from the gel onto the membrane. In the preliminary experiments, two different primary anti-ALDH3B2 antibodies were used: an affinity purified goat polyclonal antibody raised against a peptide mapping within an internal region (15–20 amino acid residues between 310 and 360 (numbering based on short ALDH3B2 isoform) of ALDH3B2 of human origin (T-13 Santa Cruz Biotechnology, sc-109919, Dallas, TX, USA) and an affinity purified rabbit polyclonal antibody raised against peptide mapping to 135–341 amino acid residues region of human ALDH3B2 (Novus Biologicals, NBP1-31716, Centennial, CO, USA). Their specificity was assessed by performing western blot analyses with recombinant ALDH1A1 and ALDH3A1 proteins that were available in our laboratory. None of these two ALDH recombinant proteins mentioned above produced bands in western blot analyses with primary antibody obtained from Santa Cruz Biotechnology (Supplementary Figure S2). Furthermore, to assess the possibility of this antibody to cross-react with ALDH3B1 protein, which could not be evaluated experimentally, the primary structures of the two ALDH3B proteins were compared revealing that within the epitope recognized by anti-ALDH3B2 antibody obtained from Santa Cruz Biotechnology sixteen amino acid residues are different for those two isozymes. That is why this antibody was used in further experiments.

Western blot analyses were also performed using other tissues homogenates such as ovaries and colons. The results of these analyses are shown in Supplementary Figure S2.

### 4.4. DNA Analysis

Genomic DNA was isolated from placenta fragments using Genomic Mini (A&A Biotechnology) and subjected to sequence analysis with the following primers:

5′ TGGAGGGTGCCCGTGAAG3′ and

5′ CCCTCCTGAAAGAGGACAGG3′

Primers were designed to amplify the sequence containing the premature stop codon. The obtained sequences were analyzed using Vector NTI Advance^TM^ 11.0 software. As a reference *ALDH3B2* sequence from NCBI (NG_012255.1) was used.

### 4.5. RNA Analysis

Placenta fragments were stored in RNAlater solution (Life Technologies) and then homogenized using PRO 200 homogenizer (PRO Scientific). RNA was isolated using EXTRACTME TOTAL RNA kit (DNA Gdańsk) according to the manufacturer’s instructions. Contaminating DNA was removed using DNA-free kit (Life Technologies). Pure RNA was subjected to reverse transcription and subsequent polymerase chain reactions using MyTaq^TM^One-Step RT-PCR kit (Bioline). Primers were designed to amplify the sequences containing the premature stop codon and their sequences were the following:

5′CAAGAAAGGGAGTGGAGGTG3′ and

5′TCCAGATGAAGACCGAGTCC3′.

RT-PCR was performed on a T100 Thermal Cycler (Bio-Rad) the following way: 1 cycle of reverse transcription at 45 °C for 20 min, 1 cycle of polymerase activation at 95 °C for 1 min, 40 cycles of subsequent denaturation at 95 °C for 10 s, annealing at 60 °C for 10 s, and extension at 72 °C for 30 s.

Sequence alignment was performed using Vector NTI Advance^TM^ 11.0 software. As a reference *ALDH3B2* cDNA sequences from NCBI (NM_001031615.1 and NM_000695.3) were used.

### 4.6. Protein Identification by UHPLC-ESI-MS/MS Analysis

In order to further verify the presence of ALDH3B2 in placenta homogenates an ultra-high performance liquid chromatography electrospray mass spectrometry (UHPLC-ESI-MS/MS) was applied. The proteins were resolved by SDS-PAGE. Gel bands, selected according to the molecular weight of ALDH3B2, were cut out and digested by trypsin (Trypsin Gold, Mass Spectrometry Grade purchased from Promega, Madison, WI, USA) according to the in-gel digestion standard protocol involving reduction and alkylation steps. Protein digestion was stopped by formic acid addition and obtained peptides were extracted from the polyacrylamide gel. Peptide mixtures were concentrated in a vacuum centrifuge and resuspended in 0.1% formic acid in water (*v/v*). UPLC-ESI-MS/MS analyses were performed using Dionex UltiMate 3000 RSLCnano system (Thermo Fisher Scientific, Waltham, MA, USA) coupled to Orbitrap Fusion Tribrid mass spectrometer (Thermo Fisher Scientific). Peptides’ samples were loaded first onto the trap column Acclaim PepMap100 C18, particle size 5 µm, pore size 100 Å, 300 µm i.d. × 5 mm (Thermo Fisher Scientific, Waltham, MA, USA) at the flow rate of 10 µl/min of water with 0.1% formic acid and desalted for 7 min. The injection volume was 20 µL. Acclaim PepMap100 C18, particle size 3 µm, pore size 100 Å, 75 µm i.d. × 50 cm (Thermo Fisher Scientific, Waltham, MA, USA) was used as an analytical column. Peptides were eluted from both columns at the flow rate of 300 nL min^−1^ with the following linear gradient: 0 min—3% B, 8 min—3% B, 120 min—70% B, 130 min—70% B, 130.5 min—3% B, and 150 min—3% B (solvent A—water/0.1% formic acid, solvent B—80% acetonitrile/20% water/0.1% formic acid). Eluting peptides were ionized in the positive ion mode with a capillary voltage of 1.6 kV and analyzed using an Orbitrap Fusion Tribrid mass spectrometer. Survey scans were recorded with the Orbitrap mass analyzer at a resolving power of 60,000 in the m/z range of 350–1600 and from each survey scan the most abundant multiply charged ions were fragmented by higher energy collisional dissociation and collisional-induced dissociation. The product ions were analyzed in the Orbitrap mass analyzer at a resolving power of 15,000. Cycle time was 3 s. After fragmentation the masses were excluded for 30 s from further fragmentation.

UPLC-ESI-MS/MS data was analyzed using Xcalibur 2.0.7 (Thermo Scientific, Waltham, MA, USA) and MASCOT 2.2.0.4. (Matrix Science, London, UK) [65]. The acquired MS/MS spectra were evaluated using UniProt database [15] to which the sequence of long ALDH3B2 isoform modified with alanine in the position encoded by premature stop codon (translation product of mRNA transcript number U37519.1 from NCBI obtained using ExPASy server [14] (https://web.expasy.org/translate/)) was added.

The MS/MS analysis was also performed for recombinant long ALDH3B2, as a control experiment, providing the successful use of MS/MS analysis to verify the presence of long ALDH3B2 isoform in placenta homogenate.

### 4.7. Bioinformatic Analyses

Translation of *ALDH3B2* mRNA transcript (number U37519.1 from NCBI, Bethesda, MD, USA) was achieved using ExPASy server [14]. The product of this translation process with alanine in the position encoded by premature stop codon was used as ‘long ALDH3B2′ in other bioinformatic analyses described below. Peptide sequences identified in the MS/MS analysis were mapped to the long ALDH3B2 isoform sequence and compared with the sequences of the corresponding peptides of ALDH3B1 (NCBI: NP_001154945.1, Bethesda, MD, USA) using Clustal Omega server [16]. Sequence alignment of all four aldehyde dehydrogenase isoenzymes belonging to the third subfamily (NCBI sequence NP_001128640.1 of ALDH3A1, NCBI sequence NP_000373.1 of ALDH3A2, NCBI sequence NP_001154945.1 of ALDH3B1 and long ALDH3B2 isoform sequence, Bethesda, MD, USA) was performed using Clustal Omega server [16]. The tertiary and quaternary structure models of long ALDH3B2 isoform were obtained using Phyre2 server [18] and Swiss Model server [19], respectively. In both cases rat class 3 aldehyde dehydrogenase template at 2.6 Å resolution was used (PDB ID: 1AD3). The confidence was 100%, coverage 95% (444 residues: 4–447) with 50% sequence identity. For the model obtained using Phyre2 software no backbone or sidechain relaxations were performed. The scientific group of Dr. Lawrence Kelley have found that using molecular dynamics to relax homology models typically worsens their accuracy in terms of superposability on the native structure. The backbone is inherited from the template based on the alignment and the sidechains are placed using the SCWRL algorithm based on a backbone-dependent rotamer library. Swiss Model uses the ProMod3 modelling engine where model relaxation is the last step. ProMod3 delegates the molecular mechanics energy calculations used for relaxation to the OpenMM library. The CHARMM27 forcefield in vacuum are evaluated and relaxed iteratively. Each iteration consists of 20 steps SD (steepest descent) and 10 steps LBFGS (Broyden-Fletcher-Goldfarb-Shanno). Iteration stops as soon as all stereo-chemical problems are resolved.

The probability of transmembrane helices was assessed for long ALDH3B2 using TMHMM Server v. 2.0 [27,28] and SOSUI server [29], the prediction of subcellular localization using WoLF PSORT server [30] and that of phosphorylation sites using NetPhos 3.1 [31].

## 5. Conclusions

To the best of our knowledge, despite the presence of premature stop codon in mRNA sequence, longer ALDH3B2 isoform is expressed in human placenta representing full-length protein. It can be formed by premature stop codon readthrough, a complex phenomenon enabling stop codon circumvention. Long ALDH3B2 isoform may be a functional enzyme, as all, highly conserved in ALDH enzyme family amino acids residues involved in substrate and cofactor binding, as well as oligomerization are present in its sequence. However, its activity should be confirmed experimentally.

## Supplementary Materials

Supplementary materials can be found at https://www.mdpi.com/1422-0067/20/24/6292/s1.

## Abbreviations

RT-PCR	Reverse transcriptase PCR
ORF	Open reading frame
IPTG	Isopropyl β-D-1-thiogalactopyranoside
SDS-PAGE	Sodium dodecyl sulfate-polyacrylamide gel electrophoresis
PVDF	Polyvinylidene difluoride
HRP	Horseradish peroxidase-conjugated
GAPDH	Glyceraldehyde 3-phosphate dehydrogenase
p0/MPZ	Myelin protein zero
L-MPZ	Large myelin protein zero
hnRNP	Heterogeneous nuclear ribonucleoprotein
Sec	Selenocysteine
