# Supplementary material for: Detection of ALDH3B2 in Human Placenta
Source: Int J Mol Sci. 2019 Dec 13;20(24):6292. doi: 10.3390/ijms20246292 (PMC6941052; doi:10.3390/ijms20246292)
Supplement: Supplementary file 1 [file ijms-20-06292-s001.pdf]

International Journal of
Molecular Sciences

MDPI

Article

The detection of ALDH3B2 in human placenta

Sylwia Michorowska[1,*], Joanna Giebułtowicz[1], Renata Wolinowska[2], Anna Konopka[3], Anna Wilkaniec[4], Paweł Krajewski[5], Ewa Bulska[3] and Piotr Wroczyński[1]

[1] Department of Bioanalysis and Drug Analysis, Faculty of Pharmacy, Medical University of Warsaw; e-ssolobodowska@wum.edu.pl, joanna.giebultowicz@wum.edu.pl, piotr.wroczynski@wum.edu.pl
[2] Department of Pharmaceutical Microbiology, Centre for Preclinical Research and Technology (CePT), Faculty of Pharmacy, Medical University of Warsaw; renata.wolinowska@wum.edu.pl
[3] Biological and Chemical Research Centre, Faculty of Chemistry, University of Warsaw; a.konopka@cnbc.uw.edu.pl, ebulska@chem.uw.edu.pl
[4] Department of Cellular Signaling, Mossakowski Research Centre, Polish Academy of Sciences; awilkaniec@imdik.pan.pl
[5] Forensic Medicine Department, First Faculty of Medicine, Medical University of Warsaw; pawel.krajewski@wum.edu.pl

* Correspondence: ssolobodowska@wum.edu.pl;

Received: 7 November 2019; Accepted: 11 December 2019; Published: 13 December 2019

Supplementary Materials:

Unedited images of the western blot analyses of placenta homogenates are shown in Figure S1.

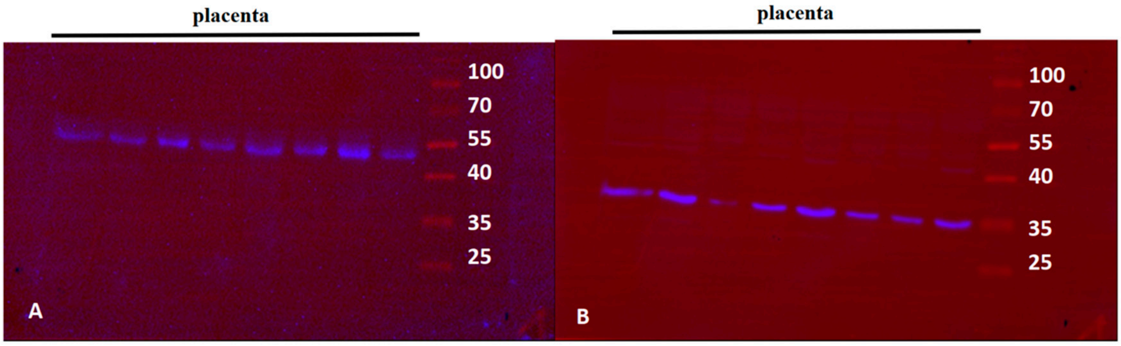

Figure S1. Western blot analysis of placenta homogenates using anti-ALDH3B2 (A) and anti-GAPDH (B) antibodies revealed bands corresponding to the molecular weight of 53 kDa (long isoform of ALDH3B2) and bands corresponding to molecular weight of 37 kDa (GAPDH). Proteins in homogenates were applied onto the polyacrylamide gel. After separation, they were transferred onto the PVDF membrane. Immunodetection of ALDH3B2 protein was performed using anti-ALDH3B2 antibody (A). After membrane stripping the immunodetection was repeated with anti-GAPDH antibody (B). GAPDH detection was used as a loading control, as GAPDH gene is constitutively expressed at high levels in many tissues. The image was taken using ChemiDoc XRS+ (Bio-Rad). As size standard PageRuler™ Prestained Protein Ladder (cat. No 26616) was used.

The images of western blot analyses of recombinant ALDH1A1, ALDH3A1 and short ALDH3B2 proteins as well as colons and ovaries homogenates are shown in Figure S2.

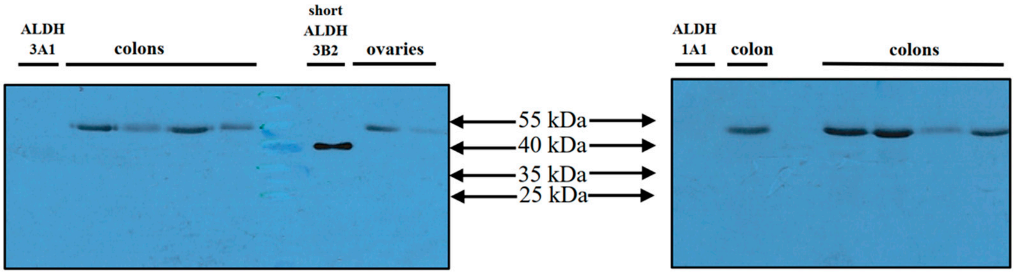

Figure S2. Western blot analyses using anti-ALDH3B2 antibody revealed bands corresponding to the molecular weight of 53 kDa (long isoform of ALDH3B2) in colons and ovaries homogenates and a band corresponding to molecular weight of 43 kDa in the case of purified short recombinant ALDH3B2. No bands were obtained in the case of purified recombinant ALDH1A1 and ALDH3A1 proteins. Homogenates and recombinant proteins were applied onto the polyacrylamide gel. After separation, proteins were transferred onto the PVDF membrane. Immunodetection was performed using anti-ALDH3B2 antibody. The chemiluminescence was detected using BioMax MR Film, Kodak.

The mass spectrum for sequencing of peptide AAQLQGLGHFLQENK found among tryptic fragments of long recombinant ALDH3B2 protein as well as sequence coverage of the recombinant long ALDH3B2 protein are shown in Figure S3.

A **Protein sequence coverage: 74%**

Matched peptides shown in ***bold red***.

1 MDPFEDTLRR LREAFNAGRT RPAEFRAAQL QGLGHFLQEN KQLLRDVLAQ
51 DLHKPAFEAD ISELILCQNE VDYALKNLQA WMKDEPRSTN LFMKLDSVFI
101 WKEPFGLVLI IAPWNYPLNL TLVLLVGALA AGSCVVLKPS EISQGTEKVL
151 AEVLPQYLDQ SCFAVVLGGP QETGQLLEHK LDYIFFTGSP RVGKIVMTAA
201 TKHLTPVTLE LGGKNPCYVD DNCDPQTVAN RVAWFCYFNA GQTCVAPDYV
251 LCSPEMQERL LPALQSTITR FYGDDPQSSP NLGHIINQKQ FQRLRALLGC
301 SRVAIGGQSN ESDRYIAPTV LVDVQETEPV MQEEIFGPIL PIVNVQSVDE
351 AIKFINRQEK PLALYAFSNS SQVVNQMLER TSSGSFGGNE GFTYISLLSV
401 PFGGVGHSGM GRYHGKFTFD TFSHHRTCLL APSGLEKLKE IHYPPYTDWN
451 QQLLRWGMGS QSCTLL

B

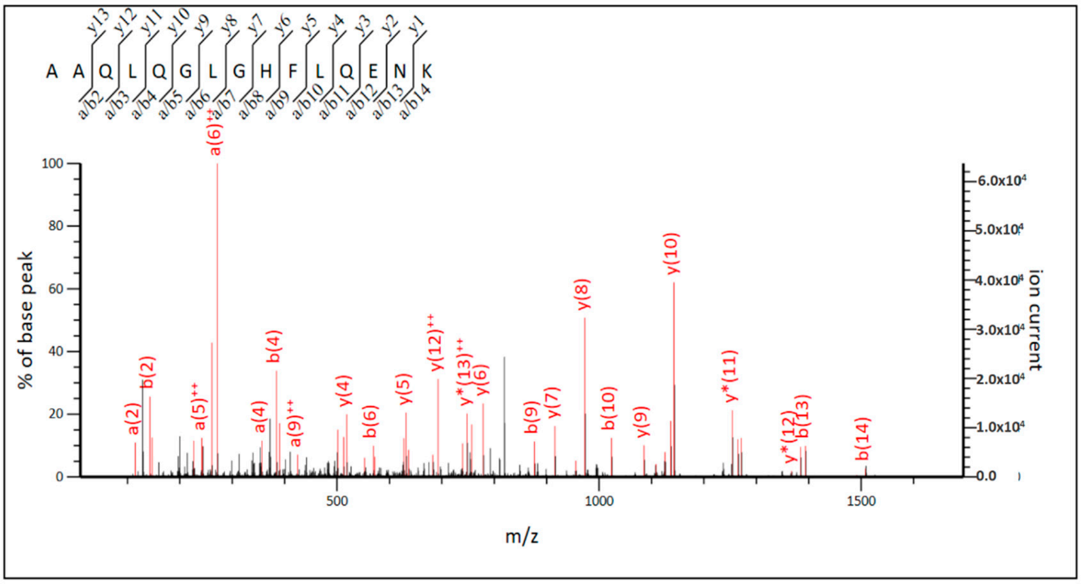

Figure S3. A. Sequence coverage of the recombinant long ALDH3B2 (from Mascot report). Peptides found experimentally are marked with red color, amino acid sequence encoded by nucleotides located upstream the second start codon is underlined, amino acid sequence of unique peptide identified also in analysis of placenta homogenate is framed with a black box. B. MS/MS spectrum registered for AAQLQGLGHFLQENK peptide. The upper left corner indicates the source of the fragment ions identified as either a, b or y ions. $^{++}$ designates doubly charged fragment ions, * designates fragment ions with neutral loss.

The probability curve for the formation of transmembrane helices by long ALDH3B2 isoform obtained using TMHMM Server v. 2.0 [1,2] is shown in Figure S4.

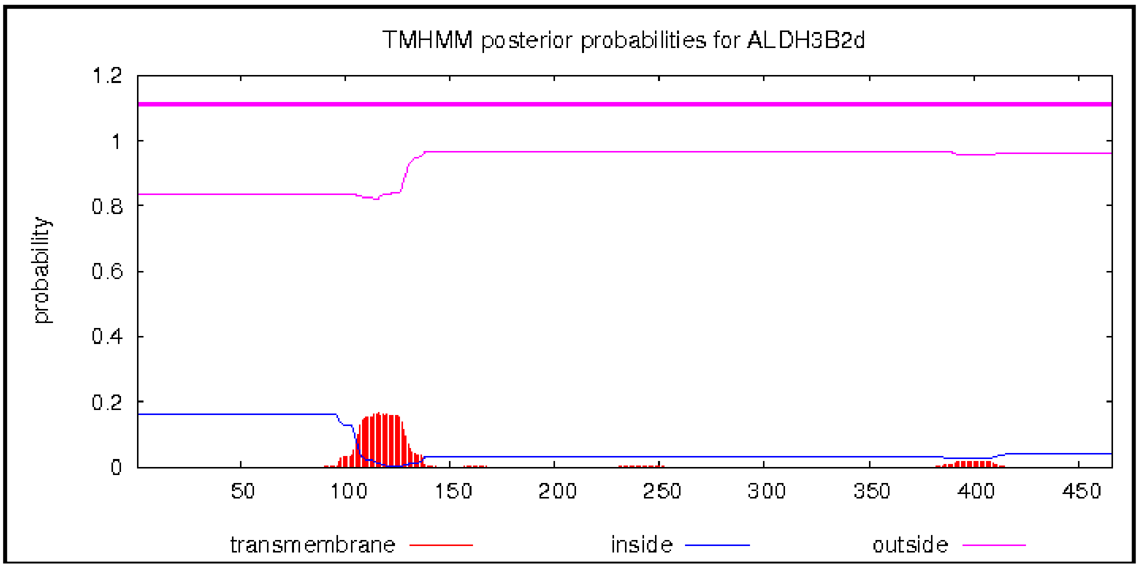

Figure S4. Prediction of transmembrane helix formation by long ALDH3B2 isoform. The probability curve for the occurrence of transmembrane helix in case of long ALDH3B2 isoform was obtained using TMHMM Server v. 2.0 [1,2] and the ALDH3B2 sequence, the product of translation of mRNA transcript number U37519.1 from NCBI obtained using ExPASy server [3] with alanine in the position encoded by premature stop codon.

The alignment of all *ALDH3B2* mRNA sequences available in NCBI is shown in Figure S5.

```
mRNA1   GTAGGAGCAGAGCCTGCGCATCTGGAGGCAGCATGTCCAAGAAAGGGAGTGGAGGTGCAG  341
mRNA2   GTAGGAGCAGAGCCTGCGCATCTGGAGGCAGCATGTCCAAGAAAGGGAGTGGAGGTGCAG  131
mRNA3   ------------------------CTGCCACCATGTGAA-G-AAGGATGTGGT--TGCTT  32
mRNA4   GTAGGAGCAGAGCCTGCGCATCTGGAGGCAGCATGTCCAAGAAAGGGAGTGGAGGTGCAG  165
mRNA5   GTAGGAGCAGAGCCTGCGCATCTGGAGGCAGCATGTCCAAGAAAGGGAGTGGAGGTGCAG  288

                                          FIRST START CODON
mRNA1   CGAAGGACCCAGGGGCAGAGCCCAC-GCTGGGGATGGACCCCTTCGAGGACACACTGCGG  400
mRNA2   CGAAGGACCCAGGGGCAGAGCCCAC-GCTGGGGATGGACCCCTTCGAGGACACACTGCGG  190
mRNA3   CCC---------------CTTCCACCATAACTGATGGACCCCTTCGAGGACACGCTGCGG  77
mRNA4   CGAAGGACCCAGGGGCAGAGCCCAC-GCTG-GGATGGACCCCTTCGAGGACACGCTGCGG  223
mRNA5   CGAAGGACCCAGGGGCAGAGCCCAC-GCTGGGGATGGACCCCTTCGAGGACACGCTGCGG  347

                    TAKE-OFF SITE PREMATURE STOP CODON          A   A  Q
mRNA1   CGGCTGCGTGAGGCCTTCAACTGAGGGCGCACGCGGCCGGCCGAGTTCCGGGCTGCGCAG  46
mRNA2   CGGCTGCGTGAGGCCTTCAACTGAGGGCGCACGCGGCCGGCCGAGTTCCGGGCTGCGCAG  250
mRNA3   CGGCTGCGTGAGGCCTTCAACTGAGGGCGCACGCGGCCGGCCGAGTTCCGGGCTGCGCAG  137
mRNA4   CGGCTGCGTGAGGCCTTCAACTGAGGGCGCACGCGGCCGGCCGAGTTCCGGGCTGCGCAG  283
mRNA5   CGGCTGCGTGAGGCCTTCAACTGAGGGCGCACGCGGCCGGCCGAGTTCCGGGCTGCGCAG  407
                                   LANDING SITE
         L  Q  G  L  G  H  F  L  Q  E  E  N  K
mRNA1   CTCCAGGGCCTGGGCCACTTCCTTCAAGAAAACAAGCAGCTTCTGCGCGACGTGCTGGCC  520
mRNA2   CTCCAGGGCCTGGGCCACTTCCTTCAAGAAAACAAGCAGCTTCTGCGCGACGTGCTGGCC  310
mRNA3   CTCCAGGGCCTGGGCCACTTCCTTCAAGAAAACAAGCAGCTTCTGCGCGACGTGCTGGCC  197
mRNA4   CTCCAGGGCCTGGGCCACTTCCTTCAAGAAAACAAGCAGCTTCTGCGCGACGTGCTGGCC  343
mRNA5   CTCCAGGGCCTGGGCCACTTCCTTCAAGAAAACAAGCAGCTTCTGCGCGACGTGCTGGCC  467

                                                        LANDING SITE
mRNA1   CAGGACCTGCATAAGCCAGCTTTCGAGGCAGACATATCTGAGCTCATCCTTTGCCAGAAC  580
mRNA2   CAGGACCTGCATAAGCCAGCTTTCGAGGCAGACATATCTGAGCTCATCCTTTGCCAGAAC  370
mRNA3   CAGGACCTGCATAAGCCAGCTTTCGAGGCAGACATATCTGAGCTCATCCTTTGCCAGAAC  257
mRNA4   CAGGACCTGCATAAGCCAGCTTTCGAGGCAGACATATCTGAGCTCATCCTTTGCCAGAAC  403
mRNA5   CAGGACCTGCATAAGCCAGCTTTCGAGGCAGACATATCTGAGCTCATCCTTTGCCAGAAC  527

                         LANDING SITE      SECOND START CODON
mRNA1   GAGGTTGACTACGCTCTCAAGAACCTTCAGGCCTGGATGAAGGATGAACCACGGTCCACG  640
mRNA2   GAGGTTGACTACGCTCTCAAGAACCTTCAGGCCTGGATGAAGGATGAACCACGGTCCACG  430
mRNA3   GAGGTTGACTACGCTCTCAAGAACCTGCAGGCCTGGATGAAGGATGAACCACGGTCCACG  317
mRNA4   GAGGTTGACTACGCTCTCAAGAACCTGCAGGCCTGGATGAAGGATGAACCACGGTCCACG  463
mRNA5   GAGGTTGACTACGCTCTCAAGAACCTGCAGGCCTGGATGAAGGATGAACCACGGTCCACG  587
```

Figure S5. Alignment of different *ALDH3B2* mRNA sequences available in NCBI. The following *ALDH3B2* mRNA sequences were aligned using Clustal Omega server [4]: mRNA1 (NCBI: U37519.1), mRNA2 (NCBI: NM_000695.3), mRNA3 (NCBI: NM_001354345.1), mRNA4 (NCBI: NM_000695.2) and mRNA5 (NCBI: NM_001031615.2). Green indicates start codons, red indicates stop codon, yellow indicates potential matching take-off and landing sites of ribosome during bypassing event and violet indicates the nucleotides encoding the peptide identified in MS/MS analysis. Its sequence is annotated above the corresponding nucleotides.

Supplementary materials can be found at www.mdpi.com/xxx/s1.

1. Krogh, A.; Larsson, B.; von Heijne, G.; Sonnhammer, E.L.. Predicting transmembrane protein topology with a hidden markov model: application to complete genomes11Edited by F. Cohen. *J. Mol. Biol.* **2001**, *305*, 567–580.
2. Sonnhammer, E.L.; von Heijne, G.; Krogh, A. A hidden Markov model for predicting transmembrane helices in protein sequences. *Proceedings. Int. Conf. Intell. Syst. Mol. Biol.* **1998**, *6*, 175–82.
3. Gasteiger, E.; Gattiker, A.; Hoogland, C.; Ivanyi, I.; Appel, R.D.; Bairoch, A. ExPASy: The proteomics server for in-depth protein knowledge and analysis. *Nucleic Acids Res.* **2003**, *31*, 3784–8.

4. Sievers, F.; Wilm, A.; Dineen, D.; Gibson, T.J.; Karplus, K.; Li, W.; Lopez, R.; McWilliam, H.; Remmert, M.; Soding, J.; et al. Fast, scalable generation of high-quality protein multiple sequence alignments using Clustal Omega. *Mol. Syst. Biol.* **2011**, *7*, 539–539.

© 2019 by the authors. Submitted for possible open access publication under the terms and conditions of the Creative Commons Attribution (CC BY) license (http://creativecommons.org/licenses/by/4.0/).
